# Assessment of Cervical Genotoxicity in Infertile Women Receiving IVF Therapy Using Micronucleus Test

**DOI:** 10.3390/jcm14196837

**Published:** 2025-09-26

**Authors:** Fatma Kılıç Hamzaoğlu, Feyza Özçelik, Kaddafi Özçelik, Ayşe Gül Zamani, Kazım Gezginç

**Affiliations:** 1 Department of Gynecology and Obstetrics, Faculty of Medicine, Necmettin Erbakan University, 42080 Konya, Türkiye; kgezginc@erbakan.edu.tr; 2 Dr. Feyza Özçelik Women’s Health Clinic, 51100 Niğde, Türkiye; feyzaozcelik@gmail.com; 3 Department of Gynecology and Obstetrics, Ömer Halis Demir Education and Research Hospital, 51100 Niğde, Türkiye; drkaddafiozcelik@gmail.com; 4 Department of Medical Genetics, Faculty of Medicine, Necmettin Erbakan University, 42080 Konya, Türkiye; agzamani@erbakan.edu.tr

**Keywords:** in vitro fertilization, micronucleus assay, cervical cytology, genotoxicity, assisted reproductive technology

## Abstract

**Background:** In vitro fertilization (IVF) has become a widely used method of assisted reproduction. However, concerns remain regarding the potential genotoxic effects of ovarian stimulation protocols used during IVF, especially in relation to cervical epithelial cells. The micronucleus (MN) assay is a validated cytogenetic biomarker of chromosomal damage and genome instability, increasingly utilized in cancer risk assessment. This study aimed to evaluate the genotoxic effect of IVF treatment on cervical epithelial cells in infertile women by comparing MN frequency before and after IVF cycles and with matched healthy controls. **Methods:** This prospective observational study included 15 women undergoing IVF and 15 age-matched healthy controls. All IVF participants had primary infertility and were undergoing their first IVF/ICSI cycle. Cervical smear samples were collected from the IVF group before and three months after treatment failure. MN assay was applied, and cytogenetic parameters (MN, binucleated cells, broken egg cells, and budding cells) were evaluated under light microscopy. Non-parametric statistical tests were used for analysis. **Results:** A statistically significant increase in MN frequency was found in the IVF group following treatment compared to pre-treatment samples and the control group (*p* = 0.001). HPV status was not assessed during the study period and is acknowledged as a key limitation. However, no significant differences were observed in other nuclear anomalies. Pre-treatment MN frequencies were not significantly different from those in controls. **Conclusions:** The findings suggest a potential cytogenetic impact of IVF-related hormonal stimulation on cervical epithelial cells, as evidenced by increased MN frequency. While no direct clinical implications were identified, these changes warrant further investigation into the long-term genomic safety of assisted reproductive technologies.

## 1. Introduction

Infertility is defined as the inability to conceive after one year of regular, unprotected sexual intercourse. It affects approximately 10–15% of couples worldwide, despite the fact that 85–90% of healthy couples are expected to achieve pregnancy within the first year. With the advancement of assisted reproductive technologies (ART), especially in vitro fertilization (IVF), the success rates of infertility treatment have increased, enabling more couples to achieve pregnancy. However, the hormonal stimulation protocols used in IVF—often involving high doses of gonadotropins—have raised concerns regarding their long-term safety, particularly in relation to potential genotoxic or oncogenic effects on reproductive tissues [1].

Cervical cancer remains one of the most common gynecologic malignancies globally, particularly in low-income settings with limited access to healthcare services and screening programs. Although persistent infection with high-risk human papillomavirus (HPV) is the primary cause, other contributing risk factors such as multiparity, immunosuppression, and hormonal exposure are under investigation. In the present cohort, HPV status was not available due to institutional practice during the study period; we therefore interpret MN findings with caution. The potential link between infertility, infertility treatments, and cervical cancer remains inconclusive. While some studies suggest that infertile women may have a higher prevalence of squamous intraepithelial lesions [2], others have reported a lower incidence of invasive cervical cancer in IVF-treated women (Yli-Kuha et al. (2012) as cited in [3]). This discrepancy underscores the need for molecular and cytogenetic markers to better understand any potential association.

The micronucleus (MN) assay is a validated and widely used biomarker for detecting chromosomal damage, genome instability, and genotoxic exposure in exfoliated epithelial cells. Its application in cervical smears has been extensively studied, particularly for cancer screening and for monitoring patients undergoing radiotherapy [4,5,6]. Furthermore, studies have shown increased MN frequency in women using hormonal intrauterine devices [7], and in animal models treated with ovulation induction agents [1], suggesting that exogenous hormonal exposure may contribute to nuclear anomalies. In light of these findings, evaluating MN frequency in cervical epithelial cells of women undergoing IVF may provide valuable insight into the cytogenetic impact of ART and help clarify any potential oncogenic risks associated with fertility treatments.

This study aims to determine whether in vitro fertilization (IVF) treatment protocols pose an increased risk of developing cervical cancer in women with infertility. To summarize the aims, this study used micronucleus (MN) assay, a specific and validated cytogenetic biomarker of chromosome damage and genotoxicity, on vaginal smear samples. Samples from IVF patients and matched control samples were compared and analyzed to determine the cytogenetic changes triggered by hormonal stimulation associated with IVF, if any, that might indicate a carcinogenic risk in the cervical epithelial tissues.

Cervical cancer, along with the associated morbidity and mortality, stands as a public health concern because of its prevalence and its wide-ranging impact on population health. It is also known to be the most common form of sexually transmitted malignancy in women. It is known to be strongly associated with high-risk strains of HPV, and the other risk factors include cigarette smoking and immunocompromised states. Perhaps the most understudied area is IVF and its infertility treatment counterpart, high dosages of gonadotropin, with the possible association to cervical cancer. This study is significant as it applies micronucleus cytological assay to evaluate cervical cytogenetic instabilities in women undergoing IVF. This might further explain the safety profile of IVF and warrant additional studies regarding the long-term oncogenic risks of assisted reproductive technologies.

## 2. Materials and Methods

This prospective observational study was conducted with the approval of the Ethics Committee of Meram Faculty of Medicine (Approval No: 2012/104, approval date: 13 April 2012). A total of 15 female patients who applied to the Department of Obstetrics and Gynecology for assisted reproductive treatment and were scheduled to undergo IVF/ICSI protocols were enrolled. All 15 IVF participants had primary infertility and underwent their first IVF/ICSI cycle. Baseline cervical smears were obtained before initiation of the long protocol. During routine pre-treatment screening, participants were assessed for STDs and symptomatic vaginal infections; individuals with suspected infection were excluded. HPV testing was not routinely performed in our clinic at that time; therefore, HPV status was not evaluated.

Detailed clinical interviews were conducted to obtain information about each patient’s medical history and prior treatments. Following general physical and gynecological examinations, necessary laboratory and imaging evaluations were performed to establish baseline reproductive and systemic health parameters. On the third day of the menstrual cycle, hormonal profiling and ultrasonographic assessment of ovarian size and antral follicle count were conducted using a 5 MHz probe (General Electric Alpha Logic 200, GE Security Inc., Coppell, TX, USA).

Ovarian suppression was achieved via a long GnRH agonist protocol. On day 21 of the cycle, 10 IU/day of subcutaneous leuprolide acetate was initiated. Downregulation was confirmed on the second day of menstruation when serum estradiol (E2) levels were below 50 pg/mL and endometrial thickness was below 4 mm on transvaginal ultrasonography. Subsequently, controlled ovarian stimulation was commenced using recombinant follicle-stimulating hormone (r-FSH) in individualized doses ranging from 150 to 450 IU/day based on age, basal FSH, and body mass index. On day 5 of stimulation, dose adjustments were made for patients whose follicle response was deemed suboptimal (follicles < 9 mm and E2 < 100 pg/mL), following a step-up protocol.

When at least three follicles ≥ 18 mm in diameter had developed, 10,000 IU of human chorionic gonadotropin (hCG) was administered intramuscularly to trigger ovulation. Oocyte retrieval was performed 36 h later under general anesthesia and sterile conditions in the lithotomy position. A 17 mm, 30 cm double-lumen aspiration needle was attached to the transvaginal ultrasound probe, and follicular aspiration was carried out under 125 mmHg negative pressure. Mature oocytes were identified and isolated, then transferred into IVF-G1 and G2 culture media.

### 2.1. Micronucleus Analysis and Control Group

The primary objective of this study was to investigate whether IVF treatment was associated with an increased risk of cervical cellular atypia using micronucleus (MN) assay as a surrogate marker. Cervical smear samples and serum estradiol levels were collected from all 15 patients both prior to the initiation of IVF and three months following treatment failure (i.e., no pregnancy achieved).

A control group of 15 healthy women aged between 20 and 40 years was also included for comparison. These participants had no history of chronic illness, medication use, radiation exposure, or smoking. Cervical smears were collected from the control group and processed under identical laboratory conditions.

### 2.2. Sample Collection and Cytogenetic Procedures

Cervical smear samples were obtained using an Ayre spatula and washed into sterile Falcon tubes containing 0.9% physiological saline. The samples were promptly transferred to the genetics laboratory on the same day. After centrifugation, the supernatant was discarded, and the cell pellet was treated with a hypotonic solution for 5 min. Following a second centrifugation, the pellet was washed twice with freshly prepared cold fixative solution (methanol:acetic acid, 3:1). Clean microscope slides were labeled with patient identification details, and the pellet was dropped onto the slides, air-dried at room temperature, and stained with 5% Giemsa for 5 min.

Each slide was examined under a light microscope at 1000× magnification (objective 100×, ocular 10×), and a total of 2000 epithelial cells were counted per sample. All smears were read by a single experienced cytologist who was blinded to group/time point, using predefined criteria (Stich & Rosin), which include smooth borders, similar or slightly reduced staining intensity compared to the main nucleus, and a size less than one-third of the main nucleus. Participants with suspected STDs/vaginal infections at screening were excluded. Only monolayer, non-overlapping, and intact cells were included in the analysis. In addition to micronucleus frequency, binucleated cells (BNC), broken egg cells (BEC), and budding cells (BC) were also quantified.

Sampling Time Points & Rationale. Pre-IVF smears were collected at baseline prior to long-protocol initiation. Post-IVF smears were obtained approximately 3 months after treatment failure notification and at least 30 days after both oocyte retrieval (OPU) and embryo transfer (ET). This 3-month window was chosen a priori to exceed the ~2–3-week epithelial turnover, minimize peri-procedural/stress-related confounding, and standardize scheduling after serum pregnancy testing. Controls were invited in the early follicular phase (cycle days 3–7) where feasible; when not feasible, cycle day was recorded. All smears were read by a single experienced cytologist blinded to group/time point.

### 2.3. Statistical Analysis

All statistical analyses were performed using the SPSS software package (Statistical Package for the Social Sciences) version 15.0 for Windows. The normality of the data was assessed using the Shapiro–Wilk test. Since the dataset did not meet the assumptions of normal distribution, non-parametric tests were applied for group comparisons. In addition to *p*-values, we report rank-biserial correlations (r_rb) for Mann–Whitney/Wilcoxon tests and Hodges–Lehmann median differences with 95% confidence intervals to aid interpretation in this pilot sample. Exploratory Spearman correlations between estradiol (E2) and MN frequency were planned a priori.

Specifically, the Mann–Whitney U test was used to compare micronucleus frequencies and estradiol levels between the patient and control groups, as well as pre- and post-treatment values within the patient group. Data were reported as median (interquartile range), and a *p*-value less than 0.05 was considered statistically significant.

Where appropriate, correlation analyses were conducted using Spearman’s rank correlation coefficient to explore the relationship between estradiol levels and MN frequency. Additionally, descriptive statistics (mean, standard deviation, median, range) were used to summarize demographic and clinical characteristics of the participants. All results were presented with corresponding confidence intervals and effect size measures where relevant to support the interpretation of the findings.

## 3. Results

This study included a total of 30 women, comprising 15 patients undergoing their first cycle of IVF treatment due to primary infertility and 15 healthy women serving as the control group. All participants were aged between 20 and 40 years, were non-smokers, and had no chronic illnesses. The demographic characteristics of the IVF group are summarized in Table 1.

Micronucleus (MN) frequency and its related cytological variations—binucleated cells (BNC), broken egg cells (BEC), and budding cells (BC)—were analyzed in vaginal smear samples collected from the IVF group before and three months after treatment and compared with the control group.

In the within-group comparison of pre- and post-IVF samples, a statistically significant increase in MN frequency was observed following IVF treatment (*p* = 0.001). However, the differences in BNC, BEC, and BC counts were not statistically significant (*p* > 0.05). These results are presented in Table 2.

When the IVF group’s pre-treatment smear results were compared with those of the control group, no statistically significant differences were found in MN, BNC, BEC, or BC values (*p* > 0.05), as shown in Table 3.

Finally, a comparison between the post-IVF group and the control group revealed a statistically significant increase in MN frequency in the IVF group (*p* = 0.001), while BNC, BEC, and BC frequencies remained statistically insignificant (Table 4).

These findings indicate that IVF treatment may be associated with an increased frequency of micronuclei in cervical epithelial cells, suggesting potential cytogenetic instability, although no corresponding increase was observed in other nuclear anomalies.

## 4. Discussion

In vitro fertilization (IVF) is a commonly used form of assisted reproductive technology (ART) that provides a means of having children for many infertile couples. On the other hand, there are some worries about its use, particularly with regard to the possible dangers of hormonal stimulation protocols that may increase the likelihood of cancer developing in the long term. Out of these worries, the possibility of IVF treatment influencing the development of cancer in the cervix still remains relatively unexplored. The goal of this research was to evaluate cytogenetic instability in the cervical epithelial cells of women undergoing IVF therapy using the micronucleus (MN) assay, a well-established form of genomic damage.

A statistically significant post-IVF increase in MN frequency was observed. Importantly, MN elevation in the absence of parallel increases in BNC, BEC, or BC does not, by itself, establish persistent DNA instability; rather, it may represent an early, potentially transient genotoxic signal associated with hormone-related proliferative/replicative stress and subsequent repair. Given the absence of HPV status—an acknowledged major determinant of cervical MN frequency—our findings should be considered hypothesis-generating. We therefore outline a follow-up design incorporating HPV genotyping and p16/Ki-67, serial MN tracking across cycles, and a larger sample size to test whether MN elevations persist or normalize.

From the results of this study, a distinct increase was apparent in MN frequency in post-IVF smear samples when compared to pre-treatment and control groups (*p* = 0.001). Gonadotropin-based ovarian stimulation appears to induce some level of chromosomal damage in cervical epithelial cells, and this stimulation may explain the observed increase. Strikingly, no significant changes were noted in other forms of nuclear anomalies such as binucleated cells (BNC), broken egg cells (BEC), or budding cells (BC). This suggests that in spite of the increase in MN, the more global indicators of nuclear instability did not increase in tandem.

The importance of MN as a predictive marker for cervical cancer has been established in the literature. Setayesh et al. (2020) [4] conducted a thorough meta-analysis which affirmed the capability of MN assays in detecting chromosomal damage in cervical neoplasia due to the sensitivity of the assay towards early genotoxic alterations. Likewise, Borges da Silva et al. (2021) and Padilha et al. (2023) [5,6] conducted MN tests on patients who underwent radiotherapy for cervical cancer and showed heightened MN frequencies as indications of therapy-related cytogenetic damage. Collectively, these studies support the assertion that heightened MN, in the absence of morphologically detectable cytological atypia, signifies greater genotoxic risk or heightened susceptibility.

However, our study differs from the research conducted by Xie et al. (2023) [2] who analyzed the outcomes of IVF in relation to cervical intraepithelial neoplasia (CIN) and posited that either CIN or its treatment could have some bearing on the success of IVF but neither focused on cytogenetic changes after IVF. In contrast, our findings emphasize the cytogenetic changes observed in cervical cells after IVF, which reflects a less mainstream area of ART safety studies.

Duran et al. (2006) [1] demonstrated the use of clomiphene citrate as an ovulation induction drug was associated with the systemic use of fertility medications because it increased the micronuclei in reticulocytes of the rats. Zamani et al. (2021) [7] implemented the MN assay with LNG-IUS users and noted an increased frequency in MN as well. These results support the premise that hormonal treatments in reproductive therapies can initiate cytogenetic alterations in cervical cells.

Following IVF procedures, MN frequency exhibited a statistically significant increase. It is questionable, however, whether the increase is associated with a higher risk of developing cervical cancer in the long term. Källen et al. and Yli-Kuha et al. [3,8] conducted large-scale studies and found that women who underwent IVF did not have a higher incidence of cervical cancer. Yli-Kuha et al. [3] even found that out of 9175 women who received IVF treatments, there was a lower rate of invasive cervical cancer as compared to the general population. These studies support that while there may be some, the hormonal changes and stimulation do not lead to transitory cytogenetic alterations.

Furthermore, the strength of the present study lies in its methodological design using within-subject paired comparisons (pre- and post-IVF), alongside the absence of the irrelevant factors of age, smoking, systemic disease, and the strict cytological criteria for MN assessment. As for other aspects of the study, there were some limitations such as accommodating only 15 participants in each group, and the follow up period which lasted three months after the first treatment failure, limiting the evaluation of the long-term impacts or the impacts over several treatment cycles of IVF.

### Limitation

This study has some limitations. First, HPV status was not available, substantially limiting causal attribution of MN changes to IVF-related hormonal stimulation. Second, the small sample size (15/15) reduces statistical power and precision; therefore, we report effect sizes with 95% confidence intervals. Third, complete matching by menstrual cycle phase in controls was not always feasible (cycle day was recorded), and the study was underpowered for phase-stratified analyses. Fourth, smears were read by a single experienced cytologist; while this ensured internal consistency, inter-observer variability could not be assessed. Fifth, the 3-month post-IVF window—chosen to exceed epithelial turnover and standardize visits—may still not perfectly capture the formation/resolution dynamics of MN. Finally, additional molecular markers (e.g., HPV genotyping, p16/Ki-67) were not assessed and should be incorporated into future studies.

## 5. Conclusions

This investigation shows a statistically relevant increase in micronucleus frequency in the cervical epithelial cells of women who underwent in vitro fertilization (IVF), indicating a potential cytogenetic effect of the ovarian stimulation protocols used. Nevertheless, no statistically relevant changes were detected in the other nuclear anomalies (BNC, BEC, BC), and the clinical significance of this cytogenetic finding is uncertain.

Based on these results, there is no direct or immediate evidence linking IVF treatment with an increased risk of cervical cancer, but there is growing concern about the genomic safety of assisted reproductive technologies (ARTs). With the increasing adoption of IVF, particularly among younger demographics, long-term, large-scale, and multi-marker studies—incorporating HPV status and other molecular indicators—are essential to assess whether the noted nuclear changes are oncogenic or a hormonal response.

In the meantime, the micronucleus assay may be effective as an initial warning indicator for cytogenetic instability in populations utilizing assisted reproductive technologies, which can aid in creating safer and more tailored fertility treatment protocols.

## Figures and Tables

**Table 1:** Characteristics of the IVF Group.

Characteristic	Mean ± SD	Minimum	Maximum
Age (years)	33.1 ± 2.0	24.0	39.0
Duration of infertility (years)	5.0 ± 2.0	2.0	9.0
BMI	24.69 ± 3.55	17.31	30.0

**Table 2:** Comparison of Micronucleus and Related Parameters in the IVF Group Before and After Treatment.

Parameter	Pre-IVF Mean Rank	Sum of Ranks	Post-IVF Mean Rank	Sum of Ranks	*p*-Value
MN	10.33	155.00	20.67	310.00	0.001 *
BNC	15.83	237.50	15.17	227.50	0.838
BEC	14.87	223.00	16.13	242.00	0.713
BC	15.50	232.50	15.50	232.50	1.000

* *p* < 0.05 statistically significant. MN: Micronucleus, BNC: Binucleated Cells, BEC: Broken Egg Cells, BC: Budding Cells.

**Table 3:** Comparison Between Pre-IVF Group and Control Group.

Parameter	IVF Group Mean Rank	Sum of Ranks	Control Group Mean Rank	Sum of Ranks	*p*-Value
MN	15.23	228.50	15.77	236.50	0.897
BNC	15.37	230.50	15.63	234.50	0.933
BEC	15.37	230.50	15.63	234.50	0.932
BC	15.50	232.50	15.50	232.50	1.000

**Table 4:** Comparison Between Post-IVF Group and Control Group.

Parameter	Post-IVF Mean Rank	Sum of Ranks	Control Group Mean Rank	Sum of Ranks	*p*-Value
MN	20.80	312.00	10.20	153.00	0.001 *
BNC	15.10	226.50	15.90	238.50	0.806
BEC	16.00	240.00	15.00	225.00	0.775
BC	15.50	232.50	15.50	232.50	1.000

* *p* < 0.05 statistically significant. MN: Micronucleus, BNC: Binucleated Cells, BEC: Broken Egg Cells, BC: Budding Cells.

## Data Availability

The data presented in this study are available on reasonable request from the corresponding author. The data are not publicly available due to privacy and ethical restrictions.

## Abbreviations

The following abbreviations are used in this manuscript

GnRH	gonadotropin-releasing hormone
IU/day	international units per day
MHz	megahertz
ICSI	intracytoplasmic sperm injection
pg/mL	picograms per milliliter
FSH	follicle-stimulating hormone
hCG	human chorionic gonadotropin
LNG-IUS	levonorgestrel-releasing intrauterine system
